# Supplementary material for: Ultra-High Dose Rate Transmission Beam Proton Therapy for Conventionally Fractionated Head and Neck Cancer: Treatment Planning and Dose Rate Distributions
Source: Cancers (Basel). 2021 Apr 13;13(8):1859. doi: 10.3390/cancers13081859 (PMC8070061; doi:10.3390/cancers13081859)
Supplement: Supplementary file 1 [file cancers-13-01859-s001.zip › cancers-1161856-supplementary.pdf]

cancers

MDPI

Supplementary Materials

Ultra-High Dose Rate Transmission Beam Proton Therapy for Conventionally Fractionated Head and Neck Cancer: Treatment Planning and Dose Rate Distributions

Patricia van Marlen, Max Dahele, Michael Folkerts, Eric Abel, Ben J. Slotman and Wilko Verbakel

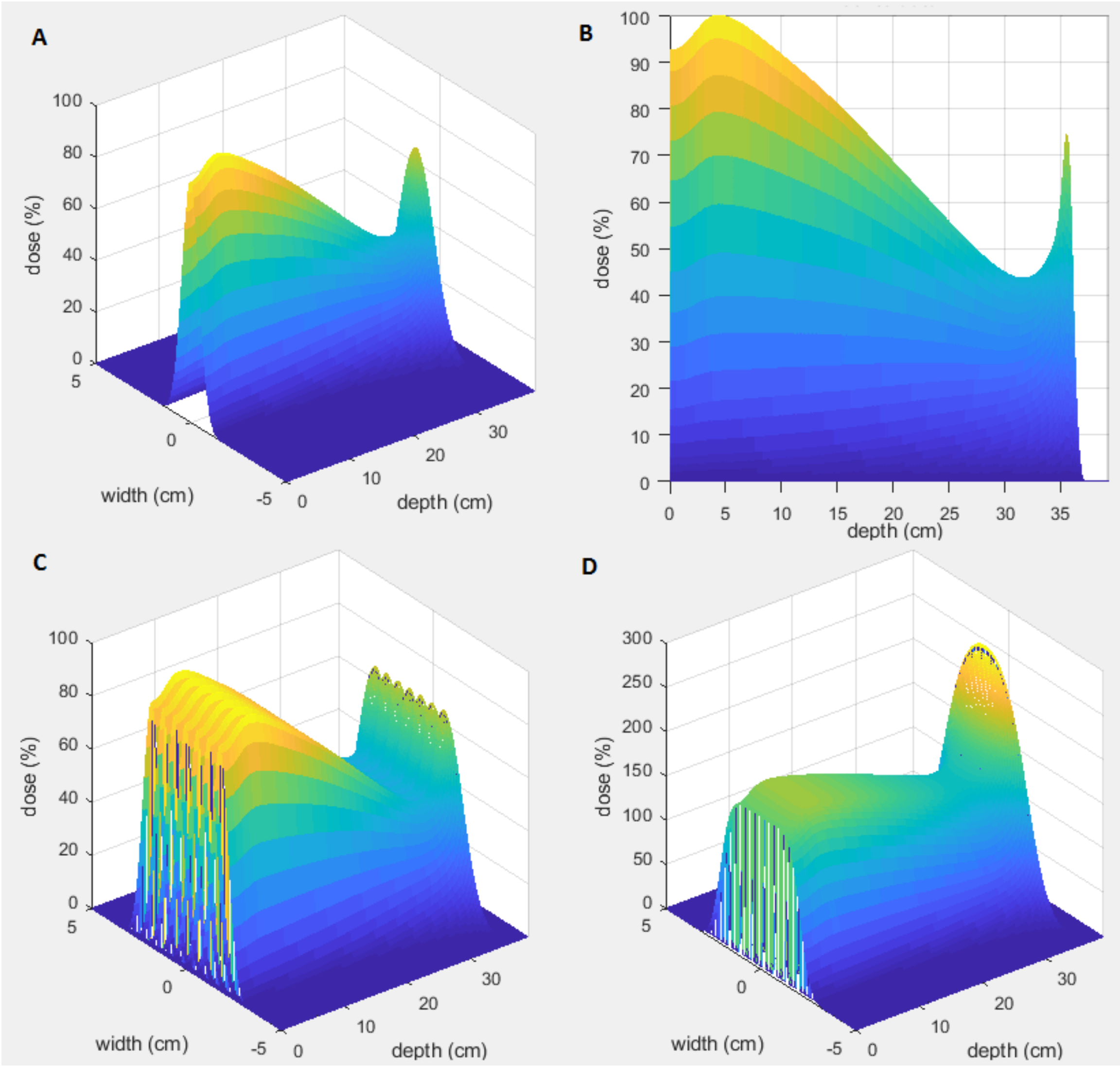

Figure S1: Dose distribution of a single 244MeV spot (A, B), using data obtained from the treatment planning system, shows the Bragg peak has a lower dose than a large part of the beam line in front of it. C, D show a multiple spot model (in 1D) and how these combined spot doses sum up to a higher total dose at Bragg peak depth. This is based on a water density phantom.

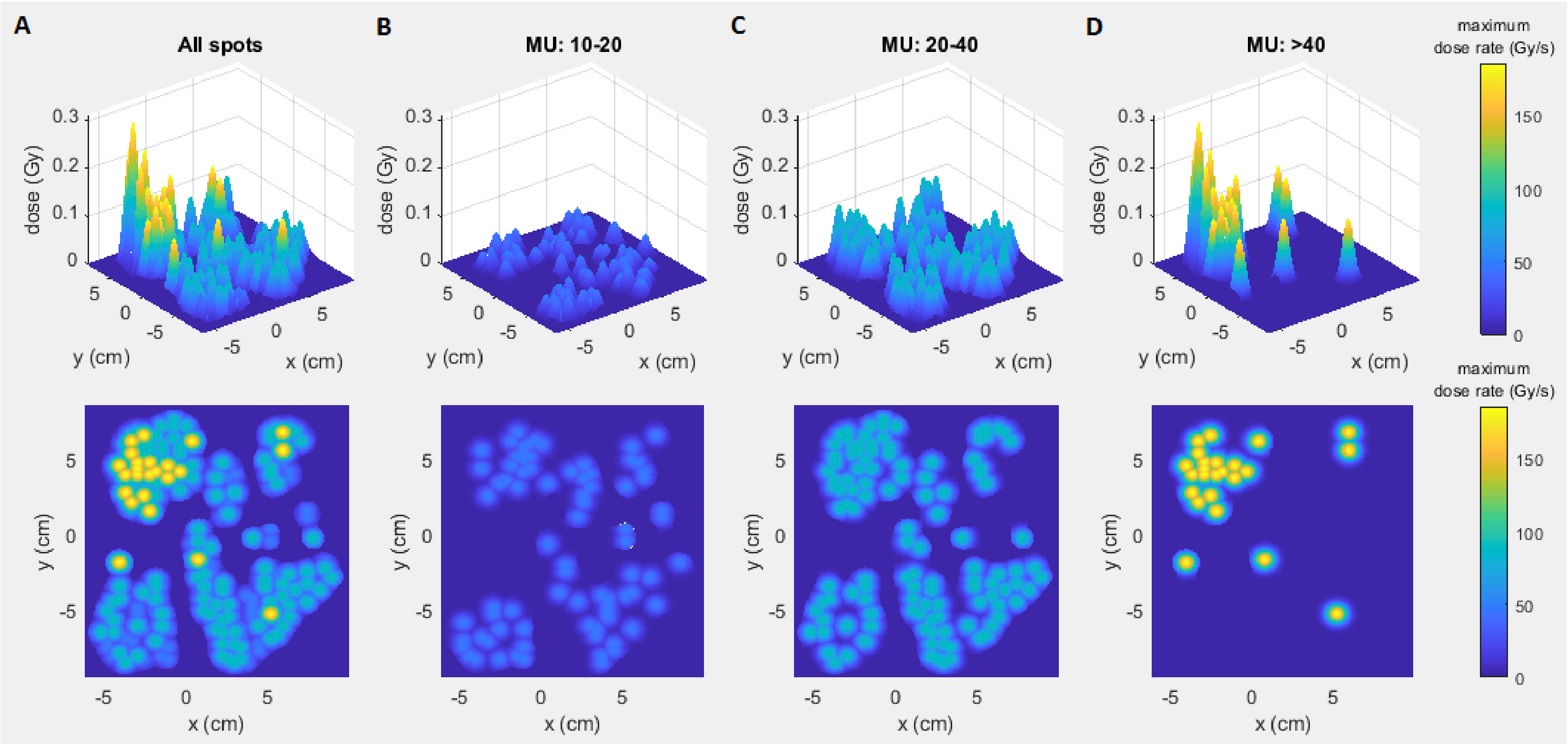

Figure S2: Side and top view of a beam cross-section. A shows the total beam and B, C, D give the spots of the subplans: 10-20 MU, 20-40 MU and ≥40 MU, respectively.

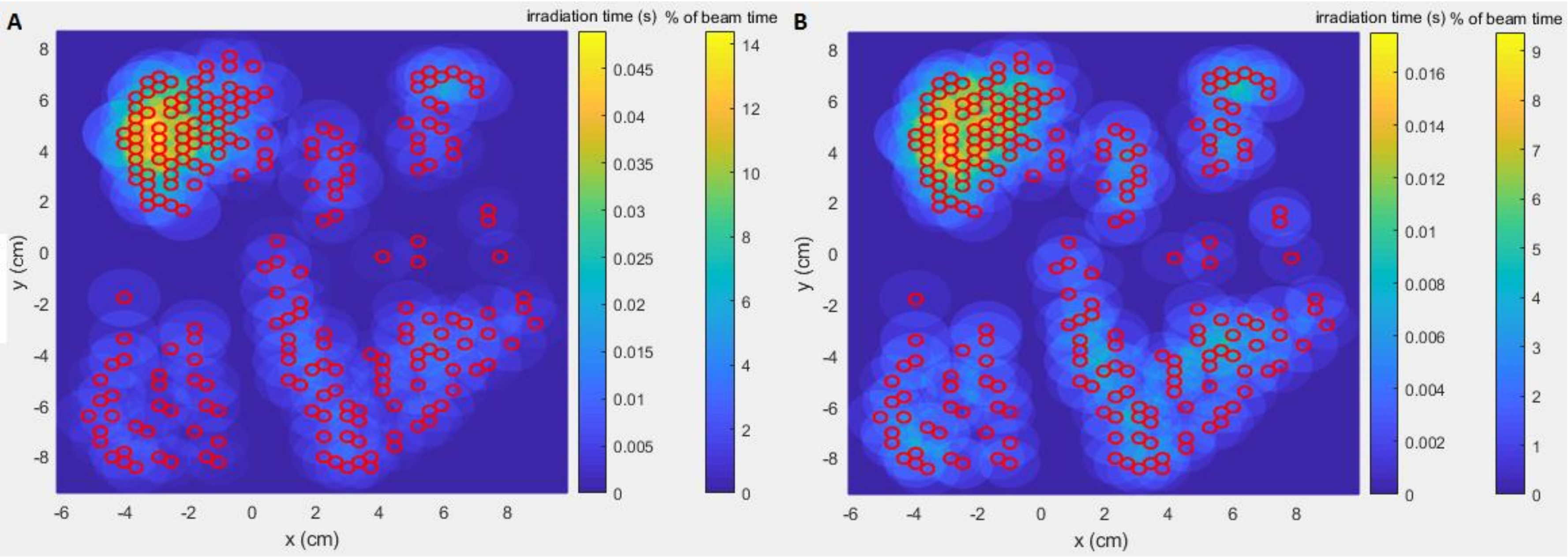

Figure S3: Time distribution within a beam for the non-split (A) and split plan (B). Red circles indicate spot locations of this beam. The irradiation time at each (x,y)-location was calculated by taking the sum of the individual spot times of spots delivering ≥0.5cGy dose at (x,y).

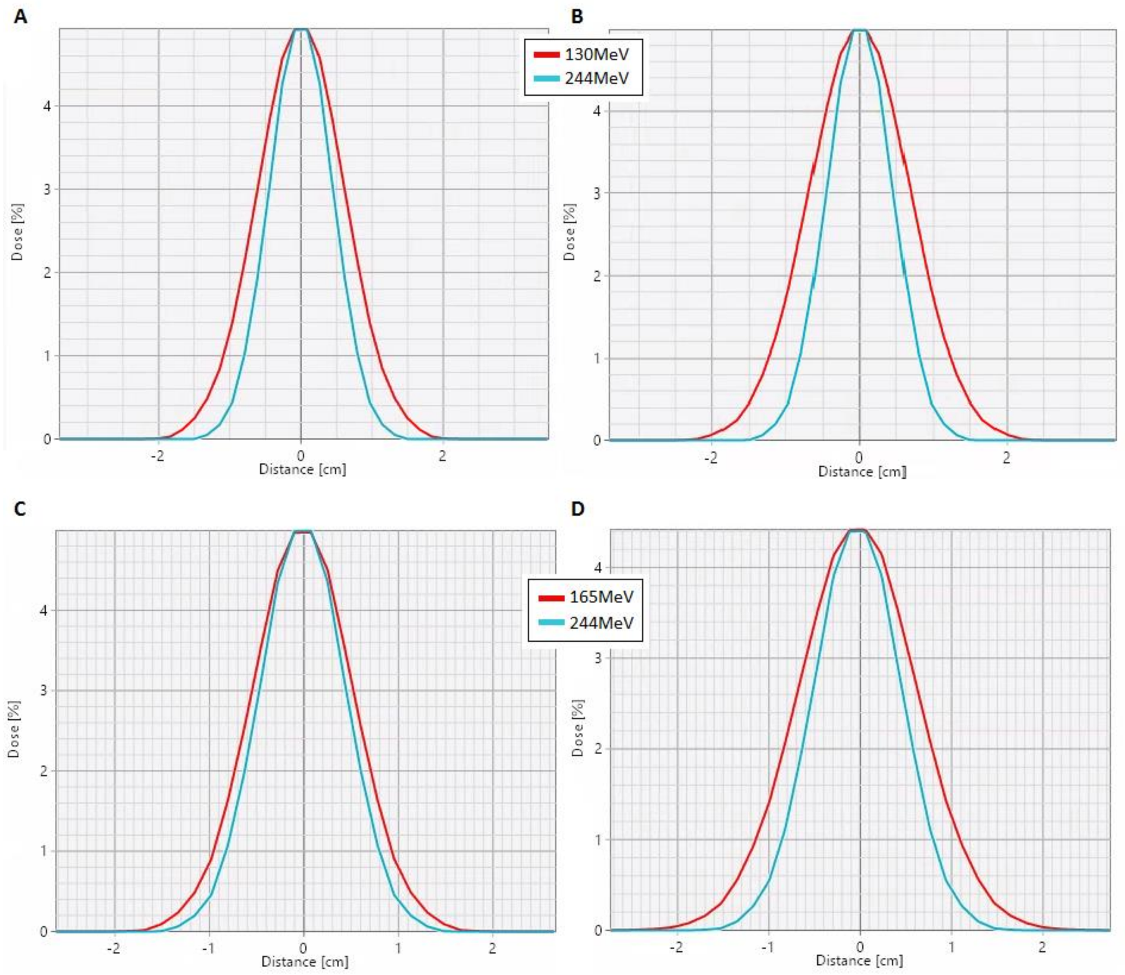

Figure S4: Penumbra comparison for 130MeV vs 244MeV in A, B and for 165MeV vs 244MeV in C, D. A and C compare at 3cm and 6cm, respectively, where both beams shows the part in front of the Bragg peak. B and D compare at 6cm and 13cm, respectively, where the lower beam energy has the Bragg peak. A 5cm range shifter is used for the 130MeV and 165MeV beams. In both cases the beam line of the 244MeV beam has a steeper penumbra.

Table S1: Average spot number and average (beam) irradiation times, both beam-on time (BO-time) and times including scanning (+S) for non-split and split plans.

	Beam 1	Beam 2	Beam 3	Beam 4	Beam 5	Beam 6	Beam 7	Beam 8	Beam 9	Beam 10	Total
# spots	118	143	235	281	336	323	283	233	138	124	2213
MU 10-20	42	51	72	86	93	88	84	72	48	44	679 (30.7%)
MU 20-40	61	75	119	144	171	163	148	117	71	65	1132 (51.2%)
MU >40	16	17	43	52	72	72	52	43	19	16	402 (18.2%)
BO-time (s)											
non-split	0.1814	0.2172	0.4001	0.4852	0.6167	0.5921	0.4877	0.4053	0.2175	0.1930	3.7962
split	0.0972	0.1177	0.1965	0.2384	0.2897	0.2754	0.2394	0.1982	0.1152	0.1030	1.8707
% difference	-46.41	-45.84	-50.90	-50.86	-53.02	-53.48	-50.92	-51.10	-47.01	-46.64	-50.72
beam time + S (s)											
non-split	0.4578	0.5500	0.8120	0.9356	1.0567	1.0403	0.9434	0.8003	0.5420	0.4751	7.6131
split	0.5493	0.6453	0.9546	1.1071	1.2102	1.1898	1.0849	0.9362	0.6285	0.5546	8.8605
% difference	19.98	17.32	17.56	18.34	14.53	14.38	15.00	16.98	15.98	16.73	16.39
